# Seed correlation analysis based on brain region activation for ADHD diagnosis in a large-scale resting state data set

**DOI:** 10.3389/fnhum.2023.1082722

**Published:** 2023-09-12

**Authors:** Tsung-Hao Hsieh, Fu-Zen Shaw, Chun-Chia Kung, Sheng-Fu Liang

**Affiliations:** ^1^Department of Computer Science, Tunghai University, Taichung City, Taiwan; ^2^Department of Psychology, National Cheng Kung University, Tainan, Taiwan; ^3^Department of Computer Science and Information, National Cheng Kung University, Tainan, Taiwan

**Keywords:** ADHD, rs-fMRI, seed-based correlations analysis, regional homogeneity (ReHo) analysis, ADHD-200

## Abstract

**Background:**

Attention-deficit/hyperactivity disorder (ADHD) is a neurodevelopmental disorder of multifactorial pathogenesis, which is often accompanied by dysfunction in several brain functional connectivity. Resting-state functional MRI have been used in ADHD, and they have been proposed as a possible biomarker of diagnosis information. This study’s primary aim was to offer an effective seed-correlation analysis procedure to investigate the possible biomarker within resting state brain networks as diagnosis information.

**Method:**

Resting-state functional magnetic resonance imaging (rs-fMRI) data of 149 childhood ADHD were analyzed. In this study, we proposed a two-step hierarchical analysis method to extract functional connectivity features and evaluation by linear classifiers and random sampling validation.

**Result:**

The data-driven method–ReHo provides four brain regions (mPFC, temporal pole, motor area, and putamen) with regional homogeneity differences as second-level seeds for analyzing functional connectivity differences between distant brain regions. The procedure reduces the difficulty of seed selection (location, shape, and size) in estimations of brain interconnections, improving the search for an effective seed; The features proposed in our study achieved a success rate of 83.24% in identifying ADHD patients through random sampling (saving 25% as the test set, while the remaining data was the training set) validation (using a simple linear classifier), surpassing the use of traditional seeds.

**Conclusion:**

This preliminary study examines the feasibility of diagnosing ADHD by analyzing the resting-state fMRI data from the ADHD-200 NYU dataset. The data-driven model provides a precise way to find reliable seeds. Data-driven models offer precise methods for finding reliable seeds and are feasible across different datasets. Moreover, this phenomenon may reveal that using a data-driven approach to build a model specific to a single data set may be better than combining several data and creating a general model.

## Highlights

- Rs-fMRI is a common approach for examining abnormal brain function in patients with psychiatric disorders. It has high potential for providing auxiliary diagnosis information through machine learning.
- The proposed data-driven strategy uses the concept of regional homogeneity to reduce the difficulty of seed selection in rs-fMRI.
- The established model was applied to a database including 73 patients with and 76 individuals without ADHD; 83.24% accuracy was achieved, and the model was unbiased.

## 1. Introduction

Attention-deficit/hyperactivity disorder (ADHD) is a common neurodevelopmental disorder in childhood and adolescence. The reported incidence of ADHD differs between both studies and countries. The estimated worldwide prevalence of ADHD is up to 7.1% in children and adolescents (Thomas et al., 2015). The Diagnostic and Statistical Manual of Mental Disorders (DSM), Fifth Edition (DSM-5) characterizes ADHD by its three cardinal symptoms: inattention, hyperactivity, and impulsivity. These symptoms affect healthy psychological functioning and are associated with mental health problems as well as impaired academic and social functioning (Missiuna et al., 2014; Asherson et al., 2016). The disorder is not limited to children and adolescents; for 40–60% of children with ADHD, the disorder persists into adulthood and results in lifelong impairment (Faraone et al., 2000; Volkow and Swanson, 2013).

Currently, the standard diagnostic procedure for ADHD is based on clinical interviews and symptom questionnaires. Although these questionnaires serve as objective and quantitative measures in conjunction with the subjective criteria listed in DSM fourth edition (DSM-4) and DSM-5, diagnosis is still time-consuming. Clinically, diagnosis requires more retrospective reports from parents and teachers than it does patient self-reports (Sibley et al., 2012). Integrating clinical questionnaires with behavioral and cognition test results is complex and challenging and requires an experienced expert evaluator (Morrill, 2009). Therefore, this process must be carefully performed by trained professionals following repeated observations and by using reports obtained from parents, teachers, or other caregivers. Moreover, the evaluator must confirm the absence of any other underlying disorders that could be mislabeled as ADHD (Rader et al., 2009). According to a survey by Morrill (2009), more than 85% of clinical staff (primary care physicians) expect auxiliary diagnostic tools for ADHD to be available. The selective use of neuropsychological tests is one such auxiliary diagnostic approach. ADHD is typically associated with substantial cognitive deficits in various neuropsychological studies, such as reaction time variability, intelligence and achievement, vigilance, working memory, and response inhibition (Pievsky and McGrath, 2018). Thus, ADHD may interfere with several brain functional structures, and this interference is reflected in behavior.

Although physiological characteristics are not included in the current diagnostic criteria, the existing body of neuroscientific evidence from structural and functional neuroimaging studies indicates a correspondence between the abnormal behaviors used for ADHD diagnosis and brain features; for example, some brain areas may have a smaller volume and be prone toward hyperactivity or hypoactivity (Konrad and Eickhoff, 2010) in patients with ADHD compared with healthy patients, such as the frontal lobe (Durston et al., 2004; Paloyelis et al., 2007), cerebellum (Semrud-Clikeman et al., 2000; Hart et al., 2012), motor cortex (Mostofsky et al., 2006; McLeod et al., 2014), and deep brain area (Schneider et al., 2006; Konrad and Eickhoff, 2010; Castellanos and Proal, 2011). To accelerate the rate at which neuroscience informs clinical practice, The ADHD-200 Consortium (2012) constructed a large-scale ADHD data set (sample size over 1,000) and a global competition to integrate information technology with brain image analysis. A survey of follow-up studies (The ADHD-200 Consortium, 2012) demonstrated that neuroimaging data, especially data from resting state functional magnetic resonance imaging (rs-fMRI), are highly promising for the diagnosis, assessment, and treatment evaluation of patients with ADHD. These objective biological tools can be used by an individual or auxiliary clinician to inform an ADHD diagnosis during their decision-making regarding treatment.

Resting state is defined as spontaneous brain activity in the absence of any specific cognitive task during the acquisition of a brain signal or image; this definition reduces the difficulty of clinical diagnosis of ADHD. Rs-fMRI has been widely applied in the diagnosis of neurological or psychiatric diseases such as ADHD, autism spectrum disorder, Alzheimer’s disease, depression, etc (Uddin et al., 2010; Lee et al., 2013; Fox et al., 2014). A variety of neurological evidence of brain network abnormalities in ADHD revealed through rs-fMRI have been reported. For example, abnormalities in default mode networks (DMN), including increases (Tian et al., 2008) or decreases in network homogeneity (Uddin et al., 2008) or decreases in connectivity (Castellanos et al., 2008; Uddin et al., 2008) in patients with ADHD were observed by using seed-based correlation analysis (SCA). Regional homogeneity analyses of rs-fMRI results have also revealed that patients with ADHD have similarly high activation in the sensory areas of the brain (Cao et al., 2006; Krain and Castellanos, 2006).

Seed-based correlation analysis (SCA) is the oldest and most popular method for investigating abnormal brain activity in rs-fMRI research on ADHD and other psychiatric disorders (Biswal et al., 1995). Due to the lack of a task design, referring the literature to selected regions of interest (ROIs) as seeds to estimate functional connectivity (FC) is common in rs-fMRI research. Because this selection usually stands with strong evidence, the results of abnormal brain investigations are more accepted and understood in clinical research. However, some seed details, such as shape, size, and boundary may induce bias in the results, and the results may be difficult to replicate or apply. By contrast, the use of general whole-brain anatomical or functional maps as seeds can increase the reproducibility of SCA results. Craddock et al. (2013) used the automated anatomical labeling (AAL) (Tzourio-Mazoyer et al., 2002), CC200, and CC400 (Craddock et al., 2012) atlases to divide the whole brain into several seeds and construct the resting networks. Regardless of the seed selection method, the auxiliary diagnostic performance reported in the ADHD-200 consortium competition (The ADHD-200 Consortium, 2012) was an accuracy of only 60 ± 5%. Thus, the model-driven seed selection method may be unsuitable for diagnosing multisymptomatic neurodevelopmental disorders. If a more flexible, data-driven seed selection method that is responsive to abnormalities in ADHD could be identified, providing auxiliary diagnostic information with SCA should become more effective.

The aim of this study is to evaluate the feasibility of diagnosing ADHD by analyzing rs-fMRI data. To achieve this goal, we propose a smart seed selection method for diagnosing ADHD in children with rs-fMRI data. We refer regional homogeneity (ReHo) analysis results. This data-driven method can determine specific seeds with within-group homogeneity and between-group differences. Furthermore, principal component analysis was used to reduce the SCA feature dimensions, and linear discriminant analysis (LDA) was used to distinguish healthy children and those with ADHD. The classification accuracies of different feature extraction approaches were also compared.

## 2. Materials and methods

### 2.1. Image data set

In this study, rs-fMRI data sets were downloaded from the ADHD-200 Global Competition.^1^ The original data sets are available on the websites of eight institutions: New York University Child Study Center (NYU), Peking University, Bradley Hospital/Brown University, Kennedy Krieger Institute, NeuroIMAGE Sample, Oregon Health & Science University, University of Pittsburgh, and Washington University in St. Louis. Except for the NYU data set, all these data sets had unbalanced data samples. For example, some centers only provided fMRI data for patients with specific ADHD subtypes. To avoid interference due to the heterogeneity of different medical centers and acquisition machines, we used only the NYU data set. The NYU data set covered 123 children with ADHD (mean age 11.2 ± 2.7 years; 97 boys and 25 girls); among these patients, 77 were diagnosed with ADHD-combined, 44 with ADHD-inattentive (AD), and two with ADHD-hyperactive (HD). The data set also included 99 controls (48 boys and 51 girls) with mean age 12.2 ± 3.1 years. To consider the reliability of classification, this study considered ADHD-combined and ADHD-inattentive patients as the ADHD group. Moreover, the following exclusion criteria were used: the image contains excessive head movement, the control group has non-ADHD psychiatric symptoms, the ADHD group has neurologic or psychiatric comorbidity (Supplementary Appendix Table 1 in Supplementary file), or data were missing for identity or diagnosis attributes. Finally, we analyzed rs-fMRI data for 73 patients in the ADHD group and 76 healthy children. There was no significant difference in age (ADHD: 11.2 ± 2.7, Normal: 11.9 ± 2.98, *t* = −1.49, *p* = 0.1365) between the two groups. The gender factor showed significant difference between the ADHD group (F/M = 16/57) and normal group (F/M = 34/42, *p* < 0.01). This study used gender as a covariate in the random sampling validation of the feature extraction.

### 2.2. Preprocessing

Stepwise structural and functional data preprocessing was previously conducted by the Neurobureau community using the Athena pipeline (Bellec et al., 2017). This pipeline comprises following steps: remove the first four echo planar imaging (EPI) volumes; perform slice time correction, realignment, and motion correction; co-register mean EPI images to corresponding anatomic image; normalize the fMRI data and mean image into the template space (NIHPD average T1 image atlases for children and adolescents 4.5–18.5 years of age published by the McConnell Brain Imaging Center were used in this study) at 4 mm × 4 mm × 4 mm resolution. Finally, we implemented bandpass filters at 0.01–0.08 Hz in the voxel time courses to exclude frequency components that were not related to resting brain activity.

### 2.3. Brain FC analysis

**FIGURE 1:**
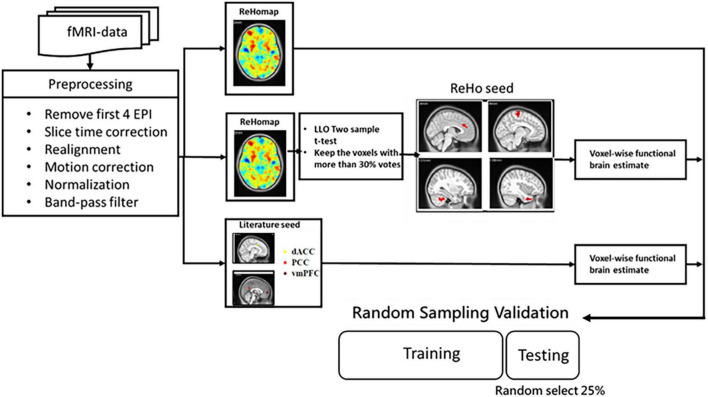
Flowchart of the proposed method of extracting rs-fMRI physiological information for auxiliary diagnosis and treatment of ADHD and non-ADHD.

The detailed architecture of the proposed approach is displayed in Figure 1. In the proposed method, a machine learning model is trained to learn FC features. Three categories of features were investigated: (a) whole-brain FC from key DMN seeds in ADHD, (b) ReHo, and (c) a hybrid of regional homogeneity and seed correlation. The performance of features and classifiers were evaluated by random sampling. In training and verification, principal component analysis (PCA) was used to reduce the dimensionality of the features.

#### 2.3.1. FC of SCA

Functional connectivity (FC)-SCA is the oldest and most popular FC analysis method in rs-fMRI research on ADHD and other psychiatric disorders. FC denote long-range correlations between seed voxels or ROIs and other individual voxels in the brain area. The selection of seeds typically has a strong theoretical basis; a seed could be a reported lesion or a hub or node in the brain network. Three seeds are often used in studies of the resting state and ADHD. In this study, we selected the dorsal anterior cingulate cortex (dACC, MNI coordinates *x* = 8, *y* = 7, *z* = 38), ventro-medial prefrontal cortex (vmPFC, MNI coordinates *x* = 2, *y* = 56, *z* = 0), and the precuneus/posterior cingulate cortex (PCC, MNI coordinates *x* = −2, *y* = −54, *z* = 27) as the seeds with a 6-mm radius. FC between each seed and the whole-brain voxels were estimated with Eq. (1). Voxel-wise correlation coefficients that indicate significant differences between groups were reserved as features for classifier training (*p* < 0.01 and cluster size ≥ 10).


(1) $$\text{CC}=\frac{\sum_{\text{i}=1}^{\text{N}}\left(\text{r}\,\left(\text{i}\right)-\bar{\text{R}}\right)*\left({\text{F}}_{\text{i}}-\bar{\text{F}}\right)}{\sqrt{\sum_{\text{i}=1}^{\text{N}}{\left(\text{r}\,\left(\text{i}\right)-\bar{\text{R}}\right)}^{2}*\sum_{\text{i}=1}^{\text{N}}{\left(\text{Fi}-\bar{\text{F}}\right)}^{2}}},$$

where CC indicates the cross-correlation coefficient between the seed and the target voxel. *r* is the reference average time course of the seed and *F* is the signal of voxels to be analyzed. $\bar{R}$ and $\bar{F}$ are the mean values of the reference and the compared time series, respectively. To eliminate individual differences, the Fisher z transformation was performed on the whole-brain FC of each individual. This study considered these three seeds as the valuable feature (literature FC, LFC).

#### 2.3.2. ReHo analysis

Regional homogeneity (ReHo) was used to measure brain intrasimilarity. If a given voxel has a high intensity, its cluster has high regional temporal synchronization. For a given voxel *p*, ReHo was defined as Kendall’s coefficient of concordance (KCC) (Kendall and Smith, 1939). The homogeneity coefficient of a time series with those of its K − 1 nearest neighbors can be used measure the similarity of multiple time series as with Eq.2:


(2) $$\text{W}\,\left(\text{p}\right)=\frac{\sum {\left({\text{R}}_{\text{i}}\right)}^{2}-\text{n}\,{\left(\bar{\text{R}}\right)}^{2}}{\frac{1}{12}*{\text{K}}^{2}\,\left({\text{n}}^{3}-\text{n}\right)},$$

in this equation, *W* is KCC among the given voxels from 0 to 1 and indicates the similarity of the activity of voxels in a cluster. *R*_i_ is the sum of the rank of the *i*th time points, $\bar{\text{R}}$ = [(*n* + 1) × K]/2 is the mean of all *R*_i_, *K* is the number of neighboring voxels plus 1 (here, *K* = 27), *n* is the rank (here, *n* = 172, the number of EPI volumes in each scan).

#### 2.3.3. ReHo seed correlation (ReHo-SC) analysis

Although relevant literature and map templates provide some guidance for seed selection, the representativeness of the selected regions for abnormalities in the ADHD brain is still controversial. If the seed is too small, the average signal is not representative; if the seed is too large, excessive useless signals are captured. In this study, we propose a novel data-driven seed selection method based on a ReHo map. After generating the ReHo map, we select the four largest clusters as seeds for the whole-brain FC calculation. The clusters (named ReHo seeds) marked as having significant differences on the ReHo map have significantly greater regional similarity among the ADHD and control groups, respectively. If the average signals extracted from two ReHo seed differ in regional representation between groups, voxel-wise FC calculations are expected to enable the generation of distinguishable features FC. To eliminate individual differences, the Fisher’s *z* transformation was performed on the whole-brain FC of ReHo seeds (ReHo-FC) of each individual.

### 2.4. Feature extraction

After feature estimation, two steps were performed to extract features for classifier training. First, the whole-brain voxel-wise two-sample *t*-test was used to determine candidate features. As suggested in AlphaSim (Ward, 2000), a cluster size threshold was set to correct the results of the *t*-test. The candidate cluster should be significant at least the *p* < 0.01 level and cover more than 10 voxels, and these voxels should be at least connected by their edges. To avoid overfitting, we also applied the leave-one-out cross-validation pipeline in a two-sample *t*-test with the gender covariate. This study would generate 149 t maps in each *t*-test procedure then found a valuable feature map through the voting procedure. The candidate voxels need to get >33% of the votes. Empirically, the study needs to reduce the order of candidate voxels before training a classifier. PCA is a technique of identifying key modes of variation in high-dimensional data as a set of orthogonal directions in space. It attempts to determine linear combinations of the original features that explain most of the variance in these features by using just a few components. In this study, for each trial (validation) of classifier training and testing, the number of the principal components (PCs) was determined as that with the best classifying performance for the training set; the search range included PCs that explained 50–90% of total variability.

### 2.5. Classification

Fisher’s LDA was used to distinguish normal and ADHD children. LDA is a well-known scheme for pattern recognition (Pereira et al., 2009). Zhu et al. (2008) also used this method for the classification of small data sets (normal individuals = 11, patients with ADHD = 9). In the method, an algorithm is trained by using samples to determine the optimal projection of the data into a lower-dimensional vector space such that the between-group distance is maximized, and the within-group distance is minimized. In this study, accuracy is evaluated by random sampling validation. We randomly selected 25% of the data as the test set, while the remaining data was the training set. This process was repeated ten times, and the average value was reported as the result. Sensitivity (SE) indicates the percentage of correctly predicted ADHD patients, while specificity (SP) indicates the percentage of correctly classified normal individuals. The method’s overall performance is assessed using total accuracy (ACC).

## 3. Results

### 3.1. Classification performance

The performance of distinguishing subjects with and without ADHD using rs-fMRI functional connection features, specifically LFC, ReHo (Zhu et al., 2008), and ReHo-FC, is presented in Table 1. The proposed ReHo-FC features achieved accuracy rates of 83.89 and 81.08%, sensitivities of 83.89 and 86.67%, and specificities of 82.63 and 75.79% for linear models (LDA and linSVM) in differentiating between individuals with and without ADHD. Overall, our study demonstrates an improvement in performance compared to using ReHo and LFC features alone. Additionally, when employing complex nonlinear classifiers (RBF-SVM, BPNN, and 2D-CNN), ReHo-FC consistently outperformed the other two types of features. We conducted experiments with different feature combinations to assess the proposed features’ complementarity further. As presented in Table 1, regardless of the classification model employed, incorporating ReHo-FC with other features resulted in an accuracy improvement of at least 5%. The overall accuracy rate exceeded 80% for all combinations. Among them, the combination of ReHo-FC and LFC exhibited the highest accuracy when using two features. The ReHo-FC features were divided based on the selected seeds, and individual linear classifiers (LDA) were constructed to assess the contribution of each ReHo seed to ADHD classification. The accuracy of each ReHo-FC seed (mPFC: 73.51%, temporal pole: 75.14%, putamen: 74.60%, motor area: 73.24%) was comparable to or higher than ReHo (73.78%), but slightly lower than LFC (79.73%).

**TABLE 1:** Classification accuracies with different features for ADHD identification.

Feature combinations				
	PCs	Sensitivity (%)	Specificity (%)	ACC (%)
**LDA**				
ReHo-FC	23.80	83.89	82.63	83.24
LFC	20.00	78.33	81.05	79.73
ReHo	4.10	76.67	71.05	73.78
ReHo-FC + LFC	13.60	85.00	84.74	84.87
ReHo-FC + ReHo	55.30	78.89	85.26	82.16
ReHo + LFC	16.10	90.00	76.84	83.24
All features	32.90	86.67	84.21	85.41
**RBFSVM**				
ReHo-FC	11.30	81.67	80.00	80.81
LFC	13.10	80.55	73.68	77.03
ReHo	9.60	68.33	63.16	65.68
ReHo-FC + LFC	48.20	90.55	83.68	87.03
ReHo-FC + ReHo	67.70	78.89	81.05	80.00
ReHo + LFC	24.60	85.00	73.16	78.92
All features	58.80	89.44	80.00	84.59
**Linear-SVM**				
ReHo-FC	23.70	86.67	75.79	81.08
LFC	13.10	80.55	76.84	78.65
ReHo	4.30	73.33	70.53	71.89
ReHo-FC + LFC	13.40	83.89	87.89	85.95
ReHo-FC + ReHo	18.70	82.78	81.05	81.89
ReHo + LFC	8.70	80.55	80.00	80.27
All features	13.60	83.89	85.79	84.87
**BPNN**				
ReHo-FC	11.50	85.56	71.58	78.38
LFC	20.20	73.89	75.26	74.59
ReHo	3.10	60.00	63.68	61.89
ReHo-FC + LFC	17.10	90.55	82.10	86.22
ReHo-FC + ReHo	67.70	76.67	78.95	77.84
ReHo + LFC	16.10	82.22	72.11	77.03
All features	13.60	88.89	83.16	85.95
**2D-CNN**				
ReHo-FC		76.32	76.67	76.49
LFC		66.84	71.11	68.92
ReHo		57.89	68.33	62.97
ReHo-FC + LFC		80.53	82.78	81.62
ReHo-FC + ReHo		72.63	84.44	78.38
ReHo + LFC		74.74	73.33	74.05
All features		84.21	86.11	85.13

LFCS, literature functional connectivity; ReHo, regional homogeneity; ReHo-FC, ReHo seed functional connectivity.

### 3.2. Patterns in NYU data set

#### 3.2.1. ReHo

**FIGURE 2:**
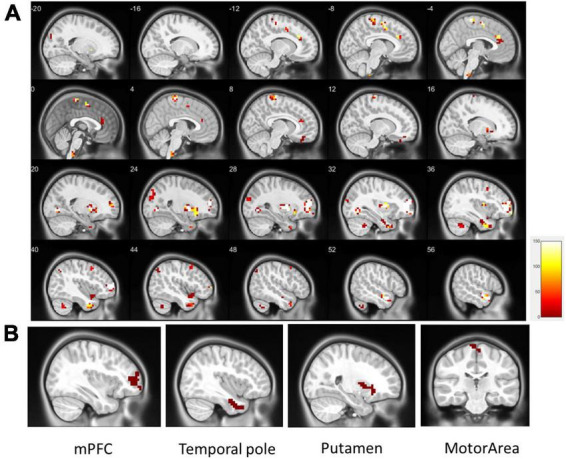
**(A)** Significant patterns in the ReHo map between the all ADHD and control groups (*p* < 0.01, got over 33% voting and with at least 10 voxels connected in the cluster); **(B)** based on the results of the one-out-two-sample *t*-test, the four largest clusters with more than 33% votes were used as ReHo seeds (clusters in the same area are not repeatedly selected). These were located in the mPFC (130 voxels), temporal pole (90 voxels), putamen (89 voxels), and motor area (28 voxels).

Regional homogeneity (ReHo) was used to estimate regional activation patterns through indices of localized concordance. On the basis of regional intrasimilarity, the ReHo method indicated that several localized areas were highly discriminative regions between the ADHD and control groups. As presented in Figure 2, abnormal regions identified by the two-sample *t*-test (*p* < 0.01, got over 33% voting and with at least 10 voxels connected in the cluster) were mostly located in the mPFC (130 voxels), temporal pole (90 voxels), putamen (89 voxels), and motor area (53 voxels). Abnormal regions were also identified in the occipital lobe and cerebellum (Table 2). Most of the abnormal brain regions had higher ReHo in patients with ADHD than in healthy individuals. However, ReHo was lower in the superior temporal and frontal lobes. Moreover, cluster coverage was used to exclude small and scattered clusters. Thus, we chose the four largest clusters (mPFC, temporal pole, putamen, and motor area) as the ReHo seeds for further estimation using ReHo-FC.

**TABLE 2:** Detailed differences of ReHo between the ADHD and control groups (sorted by cluster size).

X	Y	Z	Cluster size (voxels)	Region
32	64	−10	130	Middle frontal gyrus R
52	0	−18	90	Temporal_Pole_R
24	4	−2	89	Putamen_R
−4	−32	66	53	Paracentral_Lobule (Motor area)
−32	−4	−2	47	Putamen_L
−12	32	26	37	Anterior cingulate
−24	−84	22	34	Occipital_Sup_L
40	−60	−42	33	Cerebellum Posterior Lobe_R
−8	0	50	28	Supp_Motor_Area_L
32	−64	−6	26	Temporal Lobe_R
28	−92	22	23	Occipital_Sup_R
−64	−40	−2	20	Temporal Lobe_L
40	8	−18	16	Superior temporal Gyrus_R
−8	−36	−62	15	Cerebellum
36	8	46	13	Middle frontal Gyrus_R
−52	−64	−42	12	Cerebellum_Crus1_L
8	36	−30	10	Brodmann area 11_R
48	−72	42	10	Brodmann area 7_R

**FIGURE 3:**
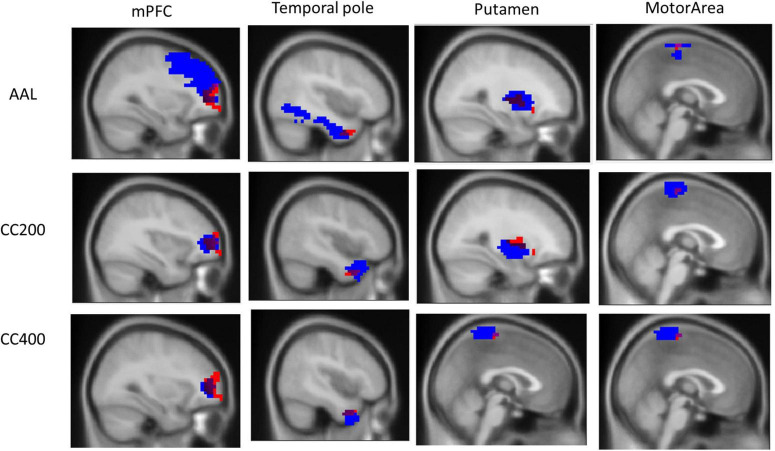
Overlap of the ReHo seed and the three atlas templates AAL, CC200, and CC400. The overlapping part covers a small portion of the AAL, CC200, and CC400 atlases templates of 6.10, 26.82, and 34.31% for the mPFC; 3.07, 13.54, and 31.81% for the temporal pole; 17.39, 9.67, and 18.70% for the putamen; and 5.28, 12.41, and 7.36% for the motor area.

However, we observed that the ReHo seed (height regional distance homogeneity) did not substantially overlap with either the anatomical or functional atlases. Figure 3 presents the overlap with the three atlases. The ReHo seed only occupied a small portion of the atlas region for mPFC (AAL: 6.10%, CC200: 26.82%, and CC400: 34.31%), the temporal pole (AAL: 3.07%, CC200: 13.54%, and CC400: 31.81%), putamen (AAL: 17.39%, CC200: 9.67%, and CC400: 18.70%) and the motor area (AAL: 5.28%, CC200: 12.41%, and CC400: 7.36%). This result implies that if the atlas is used to select the seed, the seed may contain numerous voxels that are not unrelated to the phenomenon of investigation, reducing the representativeness of the average signal.

#### 3.2.2. ReHo seed correlation

**FIGURE 4:**
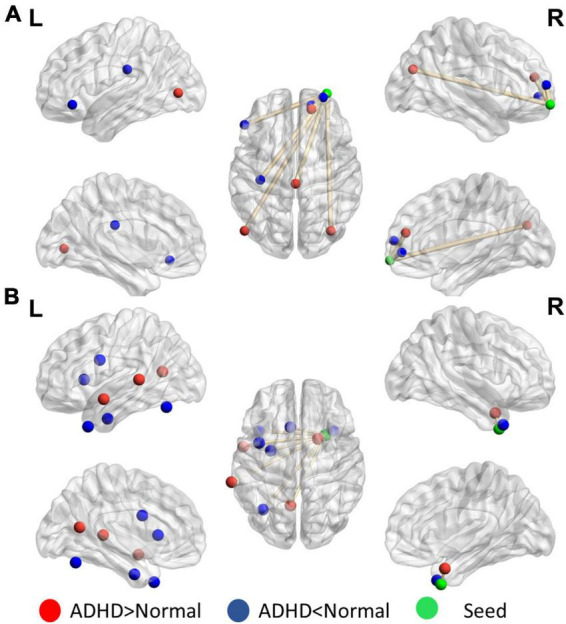
Significant patterns in the **(A)** mPFC and **(B)** temporal pole in a voxel-wise comparison of correlations between the ADHD and control groups (*p* < 0.01 and at least 10 voxels connected in a cluster with over 33% votes). Red indicates greater connections in the ADHD group than in the control group. For the mPFC, the ADHD group has stronger connections with the frontal lobe but weaker connection with the parietal and occipital lobes. The temporal pole in ADHD patients have stronger connectivity within the contralateral superior temporal lobe.

Figure 4 presents the distinguishing features of FC the significant mPFC and temporal pole ReHo-FC. Detailed regional information is presented in Supplementary Appendix Table 1. For the mPFC, a strong correlation was observed with ADHD and links in the DMN frontal hub and its subsystem covering the superior frontal gyrus and anterior cingulate cortex. However, a weak connection was observed in the links between the posterior DMN part-precuneus, temporal lobe, and occipital lobe. This result reveals that ADHD is associated with not only strong connectivity within the temporal lobe (DMN subsystem) but with strong links to the hippocampus.

**FIGURE 5:**
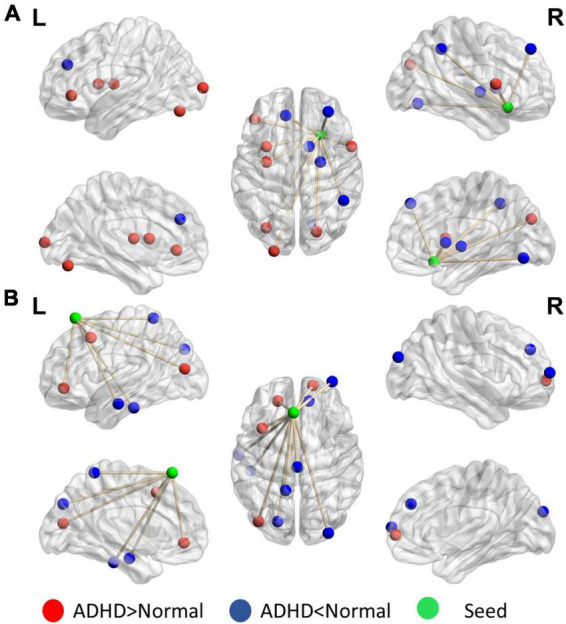
Significant patterns in the **(A)** putamen and **(B)** motor area in a voxel-wise comparison of correlations between the ADHD and control groups (*p* < 0.01 and at least 10 voxels connected in a cluster with over 33% votes). Red indicates higher connections in the ADHD group than in the control group. For the putamen, compared with the control group, the ADHD group had stronger connection with other members of the basal ganglia. In the motor area, significant differences were observed in the connections with the frontal lobe and temporal gyrus.

Figure 5 reveals that the putamen, as a part of the striatum, has multiple significant strong and weak connections (*p* < 0.01, got over 33% voting and with at least 10 voxels connected in the cluster) in children with ADHD compared with healthy controls. The detailed regional information is presented in Supplementary Appendix Table 2. These enhanced links can be divided into two categories: one was the other members of the basal ganglia (e.g., the caudate and thalamus), and the others covered the anterior cingulate cortex. By contrast, the connectivity between the putamen and occipital lobe in the ADHD group were lower than those in the healthy control group. For the motor area, strong connections were observed in the cap around the motor area for the ADHD group, but the link with the right mPFC was weak.

## 4. Discussion

At present, no ideal biological test for ADHD diagnosis exists. Currently, ADHD is primarily evaluated by observing the symptoms listed in the diagnostic criteria. The availability of more physiological information (e.g., brain images or signals) is key to improving the diagnosis or treatment of ADHD by medical teams. Such physiological information can provide objective information about cognitive deficits and increase the confidence of the medical team in diagnosis. Rs-fMRI is an excellent acquisition method for physiological brain information for people with psychiatric disorders. However, analyzing rs-fMRI data and confirming observations is challenging due to the lack of a preexisting model; in this study, we propose a procedure for automatically determining effective seeds for investigating brain abnormalities in ADHD (ReHo-FC). The procedure reduces the difficulty of seed selection (location, shape, and size) in estimations of brain interconnections, improving the search for an effective seed; Our proposed feature combination of ReHo-FC and LFC can achieve a success rate of 84.87% (LDA) for identifying patients with ADHD by 10 times random sampling of the data from 149 individuals in the NYU data set. The identified ReHo seeds have been noted in studies on ADHD. The present results are sufficient for a medical center to construct an auxiliary diagnostic information system in accordance with its needs and resources (e.g., individuals, instruments, and technicians).

As noted in the previous sections, the challenge of SCA is the difficulty of selecting or defining an effective ROI as a seed. The use of anatomical and functional atlases to select seeds and then estimating brain networks is a straightforward method of analyzing abnormal FC and resting state networks (RSNs). In this study, we compared the overlap between the identified ReHo seeds and the atlas maps. The overlapping area was only approximately 10–40% of the atlas map size. Asynchronous voxels may affect the representativeness of the extracted signal. Yao et al. (2015) demonstrated bias in RSNs from different atlas and suggested that the quantification of brain networks might be substantially affected by the selection of atlas. In an example of a use of ADHD-200 with a functional atlas in practice, Chang et al. (2012) used the CC400 atlas to estimate the whole-brain connectivity network to detect ADHD. The accuracy rates were 67% (46.9% selectivity and 82.27% specificity) and 62% (41.8% selectivity and 73.13% specificity); Dey et al. (2014) reached 64.4% (selectivity 30.66% and 84.1% specificity) by using the CC200 atlas; The proposed method, in which four precise ReHo seeds were used to estimate FC, an accuracy of 81% and balanced selectivity and specificity were achieved. Moreover, the accuracy of each seed approached or exceeded 70%—higher than whole-brain atlas FC. This result supports the introduction of a data-driven method (e.g., ReHo) to improve selection efficiency in FC analysis.

Moreover, the difficulty of using multisite data is an obstacle to identifying effective auxiliary FC patterns for diagnosis. The scanning device, parameters, and protocol may differ between sites participating in the ADHD-200. For example, individuals in the NYU data set were required to close their eyes, but this is optional for individuals in the Peking data set. Moreover, individuals are insufficiently categorized into subgroups or uncategorized in some data sets. The aggregation of data sets recorded from different institutions generates a clustered structure within the data that may cause a batch effect (Olivetti et al., 2012). The presence of batch effects conflicts with one of the basic assumptions of statistical learning theory: that data are independent and identically distributed (iid). The use of non-iid data may result in suboptimal training. During the ADHD-200 competition, most teams (including our team) used non-iid information to train a general classifier model. The final scores of most teams were approximately 60 ± 5% and biased to detecting healthy individuals. Even the winners (Eloyan et al., 2012) had a substantial bias for healthy individuals (accuracy 89%, sensitivity 21%, and specificity 94%). Beyond the competition, the consistency of abnormal functional connectivity in the ADHD-200 dataset across different data centers was investigated by Zhou et al. (2019). The results show that the abnormality of seed-based connectivity in children with ADHD was inconsistent among all datasets, including those from the same research site (but not the same equipment). This inconsistency was observed even in areas frequently reported to be associated with the default mode network (DMN). Therefore, Shao et al. (2019), Riaz et al. (2020) and Ke et al. (2022) used this independent site training method. Although the proposed classifiers and the selected sites are different, they are better than the results in the above competition in discriminating ADHD patients. Itani et al. (2018) not only developed separate classification models for different sites but also proposed a method for automatically selecting an appropriate site-specific classifier based on the similarity of the testing data for prediction. The results are also better than most research results in the above competition. In this study, we focused on the NYU data set and applied the proposed feature (ReHo-FC) with LDA model. The result could reach 83.89% in total accuracy and was balanced in terms of sensitivity (82.63%) and specificity (83.24%). Moreover, the same procedure was applied to the Peking-1 data set (other data sets have the problems of overly small or excessively unbalanced samples), and our method reached 94.71% total accuracy, 76.67% sensitivity, and 98.57% specificity. Both results were superior to above results in ADHD-200. Therefore, using multiple data sets to establish multiple independent classification models may be more suitable than establishing a general model. Alternatively, as suggested by Olivetti et al. (2012), multiple data sets could be connected by using a statistical model such as Combat (Johnson et al., 2007) and Seurat Integration (Stuart et al., 2019) to correct the batch effect.

Another approach, deep learning, is usually presented as an end-to-end learning process. Deep learning can alleviate feature engineering requirements and reduce domain knowledge requirements. Its multilayer, nonlinear learning strategy provide more flexibility in features extraction and classification. Hence, deep learning is a promising approach for informaticists in abnormal brain investigation. In the practical application part of the ADHD-200, most studies have used general atlases to generate whole-brain FC features for the deep learning model. The approximate classification accuracies of the general deep learning architecture were approximately 60–75% (Riaz et al., 2017, 2018; Zou et al., 2017; Chen et al., 2020). Mao et al. (2019) skipped feature extraction and directly imported overall rs-fMRI images to construct customized long short-term memory (LSTM) and four-dimensional convolutional neural network (CNN) classification models. However, the accuracy rates (68.8 and 71.3%) were not substantially better than the aforementioned FC-based models. Thus, we propose using an FC feature calculation method with effective guidance (ReHo seed) and deemphasizing the customization of classifiers. As presented in Table 3, the proposed method is superior for distinguishing patients with ADHD. An accuracy of 81.8% can be achieved with simple linear classification. With the application of a two-dimensional CNN architecture, the accuracy rate can be increased to 82.5%. We believe that this large performance improvement compared with previous models is primarily due to reducing the size of the input feature space by identifying meaningful features. Although deep learning is a powerful tool in processing high-dimensional data, its use with MRI images is typically hindered by the relatively small size of available samples (Mao et al., 2019). The sample size required to successfully train a deep learning model depends on multiple factors, such as the type of data, network size and architecture, type of stochasticity in the data, dimensionality of the feature space, regularization schemes, and the actual target function that the deep learning neural network is intended to learn, to name only a few (Koppe et al., 2021). A systematic review even notes that, for sample sizes less than 200, deep learning models may have heterogeneous performance results (Quaak et al., 2021). The advantages of deep learning in terms of dimensionality reduction instead resulted in overfitting in a small sample data set. Because deep learning relies on multiple hidden layers, clinical staff would have difficulty adjusting the architecture to avoid overfitting and to fit their data set. Moreover, the deep learning outcome typically lacks interpretable auxiliary information. The decision process of deep learning models is also difficult to understand and visualize for human beings. Therefore, we do not recommend directly importing deep learning architectures for auxiliary diagnosis and treatment of ADHD at this stage. We are more inclined to use meaningful and well-known features in the clinic, and the comparatively simple and easily adjustable machine learning models could provide auxiliary diagnostic information.

**TABLE 3:** Performance and characteristics of other deep learning methods for comparison.

Methods	Feature type and counts	Accuracy	
		NYU	Muti-site
FCNet (Riaz et al., 2017)	Whole-brain atlas FC (over 5,000)	58.5%	
3D-CNN (Zou et al., 2017)	Whole-brain atlas FC (over 5,000)	70.5%	
Deep fMRI (Riaz et al., 2018)	Whole-brain atlas FC (over 5,000)	73.1%	
3DCNN + LSTM (Mao et al., 2019)	Overall rs-fMRI slice image (each image over 20,000 voxels)		68.8%
4D-CNN (Mao et al., 2019)	Overall rs-fMRI slice image (each image over 20,000 voxels)		71.3%
**Our proposed**			
LDA	ReHo-FC (approximately 13PCs)	83.24%	-
2D-CNN	ReHo-FC (approximately 1,690 features)	76.49%	-

Although the reasons for abnormal RSNs in ADHD are unclear, recent studies in neuroscience have identified a number of abnormal RSNs, including the DMN, ventral attention, dorsal attention, and the frontal and motor networks in patients with ADHD (Castellanos and Proal, 2011). We used a number of ReHo-FC features that belong to a DMN subnetwork (e.g., from the mPFC to the precuneus or anterior temporal lobe network or from the motor area to the mPFC). We also observed some potential connections, such as in the frontal-striatal and frontal-striatal-cerebellar circuits of the putamen, through the ReHo-FC analysis. These circuits have been frequently reported as being associated with ADHD in recent years (Krain and Castellanos, 2006; Vaidya and Stollstorff, 2008; Durston et al., 2011; Cubillo et al., 2012; Kucyi et al., 2015). These circuits enables changes in behavior according to environmental demands and can be considered to affect cognitive or behavioral control (Durston et al., 2011). Circuit hyperactivation causes children with ADHD to be unable to avoid distractions while performing a task, efficiently manipulate information in working memory, and switch between tasks or priorities (Castellanos et al., 2006). However, the result that almost 70% accuracy in ADHD classification was achieved when the circuit was observed in the task-free resting state is intriguing. We surmise that structural changes in myelin and gray matter revealed by diffusion tensor imaging caused this abnormal functional connectivity (Liston et al., 2011).

This study may have a limitation in the generalization for classification of ADHD because we excluded subjects of a small sample size (e.g., The Supplementary Appendix Table 3 presents small population of non-ADHD psychiatric symptoms in the control group and neurologic or psychiatric comorbidity in the ADHD group). We need more data with large sample size to help classification of various ADHD subtypes. Structural and functional MRI researches have revealed that various comorbidities may be involved in the pathology of ADHD (Schneider et al., 2006; Singh et al., 2006; Wolosin et al., 2009). The contribution of comorbidity on classification of ADHD subtypes remains to further investigation.

In the future, clinical auxiliary diagnosis information could be adapted to various ADHD conditions such as subtypes, comorbidities, and even age (a critical improvement in the DSM-V standard) to enhance evaluator confidence in diagnosis. Second, although our proposed features can achieve good classification rates with the NYU (83%) and Peking (94%) data sets, the deleterious effects of using multisite data sets cannot be ignored. At present, each data set is used independently, which enables the extraction of numerous meaningful features and results in an improved the accuracy rate. We expect the system to undergo more substantial development at various hospitals and pediatric centers. Finally, by integrating the models of multiple independent centers with methods such as reinforcement learning, transfer learning, bagging, and bootstrapping, developers can increase the clinical confidence in the auxiliary medical information provided by the method.

## Data availability statement

Publicly available datasets were analyzed in this study. This data can be found here: ADHD-200.

## Ethics statement

Ethical review and approval was not required for the study on human participants in accordance with the local legislation and institutional requirements. Written informed consent from the participants’ legal guardian/next of kin was not required to participate in this study in accordance with the national legislation and the institutional requirements.

## Author contributions

T-HH completed the writing of the manuscript and the summary of all the data. C-CK and F-ZS provided rs-fMRI analysis. S-FL provided the machine learning analysis. All authors contributed to the article and approved the submitted version.

## Footnotes

1
http://fcon_1000.projects.nitrc.org/indi/adhd200/
