# Supplementary material for: Seed correlation analysis based on brain region activation for ADHD diagnosis in a large-scale resting state data set
Source: Front Hum Neurosci. 2023 Sep 12;17:1082722. doi: 10.3389/fnhum.2023.1082722 (PMC10520784; doi:10.3389/fnhum.2023.1082722)
Supplement: Supplementary file 1 [file Data_Sheet_1.docx]

# Appendix

Appendix Table 1 Detailed regional information of the mPFC and temporal pole ReHo-SCs analysis for the ADHD and control groups

| ReHo Seed | X | Y | Z | Cluster_size  (4 × 4 × 4 mm^3^) | Region |
| --- | --- | --- | --- | --- | --- |
| mPFC | -44 | -36 | -34 | 16 | Temporal Lobe_L |
|  | -60 | -8 | -14 | 16 | Temporal Lobe_L |
|  | -8 | 16 | 18 | 301 | Frontal Lobe with Anterior Cingulate |
|  | -20 | 48 | -2 | 19 | Medial Frontal Gyrus |
|  | 40 | 56 | 14 | 17 | Middle Frontal Gyrus |
|  | -20 | -84 | 42 | 63 | Occipital_Sup_L |
|  | -44 | -80 | 10 | 10 | Occipital_Mid_L |
|  | 20 | -80 | 38 | 80 | Occipital_Sup_R |
|  | 28 | 32 | 30 | 26 | Frontal_Mid_R |
|  | 16 | 44 | 42 | 22 | Frontal_Sup_R |
|  | -8 | -44 | 66 | 10 | Precuneus_L |
| Temporal Pole | 32 | 8 | -42 | 52 | Temporal_Pole_Mid_R |
|  | -36 | 16 | -38 | 43 | Temporal_Pole_Mid_L |
|  | -20 | -8 | -22 | 21 | Hippocampus_L |
|  | 12 | -8 | -22 | 37 | ParaHippocampal_R |
|  | 52 | 12 | -18 | 45 | Temporal_Pole_Sup_R |
|  | -64 | -12 | -6 | 27 | Temporal_Mid_L |
|  | -56 | -32 | 2 | 24 | Temporal_Mid_L |
|  | 4 | -56 | 18 | 22 | Precuneus_R |

Appendix Table 2 Detailed regional information of the putamen and motor area ReHo-SCs analysis in the ADHD and control groups

| ReHo Seed | X | Y | Z | Cluster_size  (4 × 4 × 4 mm^3^) | Peak *t* value | Region |
| --- | --- | --- | --- | --- | --- | --- |
| Putamen | 40 | -76 | -22 | 10 | -3.327 | Cerebelum_Crus1_R |
|  | -40 | 40 | -10 | 21 | 4.326 | Middle Frontal Gyrus_L |
|  | -16 | 4 | -2 | 41 | 3.646 | Putamen_L |
|  | 20 | -72 | -6 | 15 | -3.45 | Lingual_R |
|  | 16 | 8 | 6 | 22 | 3.399 | Caudate_R |
|  | 8 | -16 | 2 | 11 | 3.425 | Thalamus_R |
|  | -28 | -88 | 14 | 75 | -4.326 | Occipital_Mid_L |
|  | 28 | -8 | 10 | 16 | 4.257 | Putamen_R aal |
|  | 24 | -72 | 26 | 136 | -4.113 | Occipital_Sup_R |
|  | -4 | 16 | 18 | 11 | 3.481 | Corpus Callosum |
|  | 32 | 36 | 26 | 24 | 3.332 | Frontal_Mid_R |
|  | -4 | 40 | 34 | 19 | 3.674 | Frontal_Sup_Medial_L |
|  | 52 | -44 | 34 | 52 | 3.767 | Supramarginal Gyrus |
|  | 48 | 20 | 38 | 19 | 3.441 | Frontal_Mid_R |
| Motor cortex | 36 | 56 | 14 | 29 | -4.074 | Middle Frontal Gyrus |
|  | -52 | -68 | 6 | 17 | 3.66 | Middle Temporal Gyrus |
|  | -52 | 32 | -2 | 17 | 3.911 | Inferior Frontal Gyrus |
|  | 12 | 52 | -6 | 16 | -4.047 | Frontal_Med_Orb_R |
|  | 0 | -28 | 62 | 10 | 3.189 | Paracentral_Lobule_L |

Appendix Table 3 the population of comorbidities or mental disorders in NYU dataset

| comorbidities or mental disorders | ADHD (Observations) | Normal (Observations) |
| --- | --- | --- |
| Adjustment disorder | 3 |  |
| Oppositional defiant disorder, ODD. | 6 |  |
| Anxiety | 4 | 6 |
| Autistic traits | 1 |  |
| depression / MDD. | 8 |  |
| social anxiety disorder | 1 |  |
| specific phobia | 4 |  |
| Central Auditory Processing Disorder. | 1 |  |
| Dysgraphia. | 4 |  |
| Subthreshold with any mental disorder | 5 | 6 |
| Tourette Syndrome synptoms, Tics. | 2 |  |
| Enuresis. | 1 |  |
| Subthreashhold in inattentive / hyperactivity |  | 1 |
| Irritability | 1 |  |
